# Time-Dependent Increase in Network Response to Stimulation

**DOI:** 10.1371/journal.pone.0142399

**Published:** 2015-11-06

**Authors:** Franz Hamilton, Robert Graham, Lydia Luu, Nathalia Peixoto

**Affiliations:** 1 Department of Electrical and Computer Engineering, George Mason University, Fairfax, VA, United States of America; 2 Department of Bioengineering, George Mason University, Fairfax, VA, United States of America; University of Michigan, UNITED STATES

## Abstract

*In vitro* neuronal cultures have become a popular method with which to probe network-level neuronal dynamics and phenomena in controlled laboratory settings. One of the key dynamics of interest in these *in vitro* studies has been the extent to which cultured networks display properties indicative of learning. Here we demonstrate the effects of a high frequency electrical stimulation signal in training cultured networks of cortical neurons. Networks receiving this training signal displayed a time-dependent increase in the response to a low frequency probing stimulation, particularly in the time window of 20–50 ms after stimulation. This increase was found to be statistically significant as compared to control networks that did not receive training. The timing of this increase suggests potentiation of synaptic mechanisms. To further investigate this possibility, we leveraged the powerful Cox statistical connectivity method as previously investigated by our group. This method was used to identify and track changes in network connectivity strength.

## Introduction

*In vitro* neuronal cultures as an experimental model of the *in vivo* brain has played a pivotal role in understanding the dynamics of neuronal networks during development as well as in response to electrical and pharmacological stimulation. Specifically, the effects of electrical stimulation on these neuronal cultures have been well documented as it pertains to studies investigating network control [1] and influences on network firing patterns [2–5].

Additionally, electrical stimulation has been utilized in a variety of studies examining the capability of *in vitro* networks to exhibit characteristics of *learning*, see for example [6–12]. Learning in these networks can be thought of as stimulus recognition whereby after some defined stimulation protocol the dynamics of the network response to the stimulus changes in a consistent manner. Once this desired response is reached, the network is said to have learned.

Building on these ideas in Ruaro et. al. [13], the authors suggested that *in vitro* cultured networks could be used as a tool for image processing based on the cultures’ ability to discriminate between different spatial configurations of stimulating electrodes. By delivering a targeted training signal to networks of hippocampal cells, they were able to show an increase in network response to certain spatial stimulation patterns which the authors hypothesized was the result of induced network potentiation.

In this paper we examined the effects of the high frequency training signal as described in [13] on networks of cortical neurons plated on microelectrode arrays. As a means of controlling for natural fluctuations in network firing dynamics, we introduced an additional group of networks that underwent a sham training period during which no training was administered. This allowed us determine whether any changes in network response dynamics was the result of the training signal or the result of network nonstationarity.

Our results indicate that the overall network response to a low frequency probing stimulation pulse was significantly enhanced for networks that received training. These results corroborate those found in [13] for hippocampal cultures. However, we also found a statistically significant time-dependent difference between trained and control networks. Post-hoc statistical analysis revealed that trained networks had an increased network response 20–50 ms after stimulus, suggesting potentiation of a synaptic mechanism.

To further probe the possibility of synaptic potentiation, we implemented a connectivity analysis on spontaneous network activity before and after training. Using the Cox statistical connectivity method [14, 15], we were able to track changes in network connection strengths resulting from the training process. We found numerous connection parameters whose strength significantly changed after training, further supporting the idea of a substantial change in the network dynamics.

## Materials and Methods

### Cell Culturing on Microelectrode Arrays

**Fig 1:**
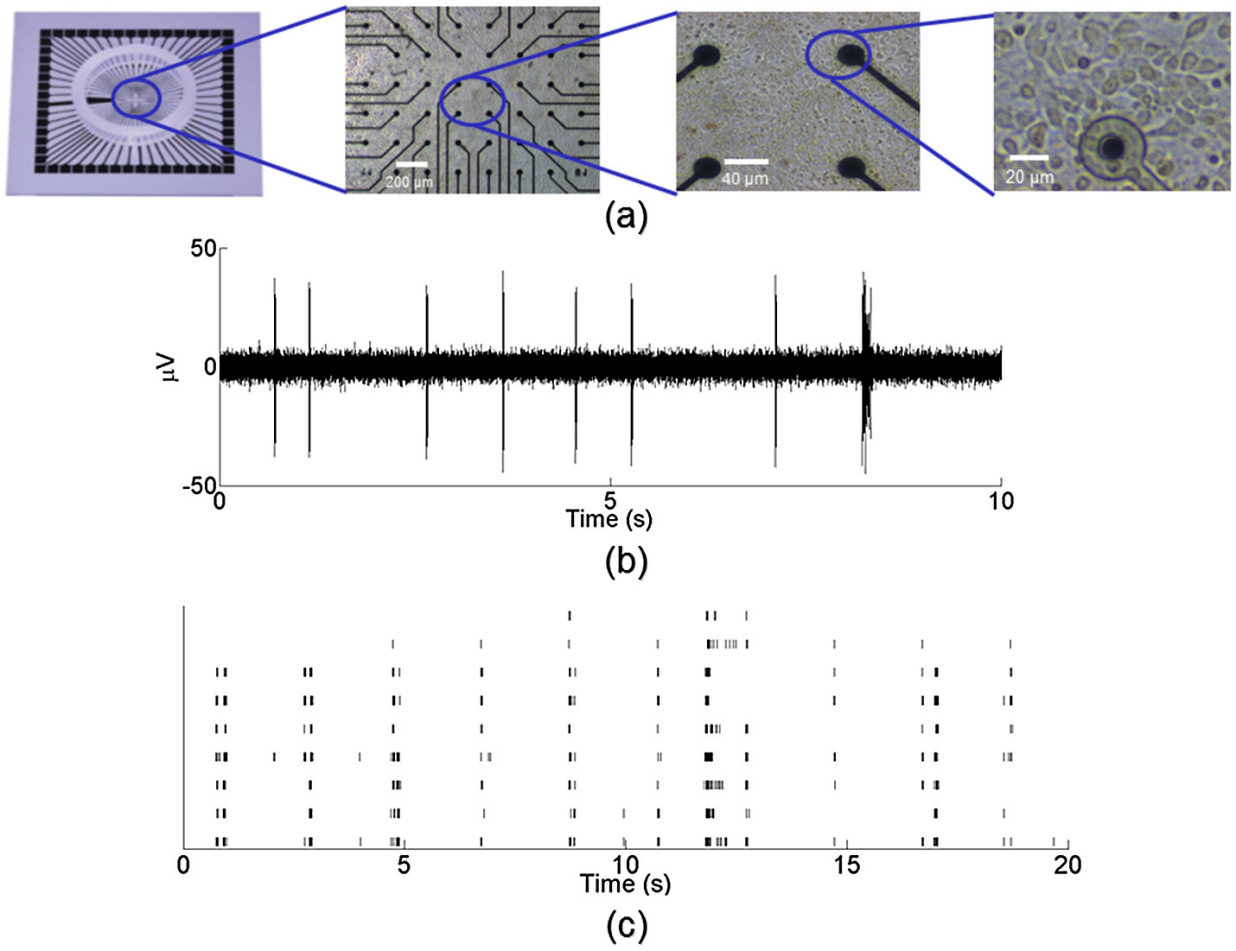
*In vitro* cultures plated on microelectrode arrays are spontaneously active. (a) Cortical neurons extracted from embryonic mice were dissociated and plated on 64-channel MEAs. MEAs allow for the simultaneous recording of the neuronal potential across the network from each electrode. (b) Extracellular potential recorded from an active electrode in a cultured network. Signal was acquired at a rate of 25 kHz and bandpass filtered from 300 Hz to 3 kHz. (c) Raster plot of twenty seconds of spontaneous activity from nine electrodes in a representative MEA network. Black lines indicate detected neuronal activity. Thresholds for spike detection were set to 5 standard deviations of the base electrode noise level.

All experiments and animal procedures were approved by George Mason University’s Institutional Animal Care and Use Committee (IACUC) under protocol #0303. Cortical neurons were extracted from E17 ICR mice. After enzymatic and mechanical dissociation, cells were plated on 64-channel microelectrode arrays (MEAs) at a density of approximately 150,000 cells per array. Further details of the culturing procedure can be found in [16]. Cultures were maintained by a 50% media exchange twice a week, and kept incubated under controlled temperature (37°C) and humidity (10% CO_2_) until experimentation at 28 days *in vitro* (DIV) or older. Fig 1a shows an example of a cultured MEA neuronal network at 28 DIV.

### Extracellular Recordings

MEAs allow for simultaneous recording of neuronal extracellular potential at each of the array’s electrodes. Cultures were hooked up to a Multichannel Systems recording system (Reutlingen, Germany) and temperature was maintained at 37°C through a temperature controller (TC02 Temperature Controller Multichannel Systems, Reutlingen, Germany). Signals were acquired at a rate of 25 kHz and bandpass filtered from 300 Hz to 3 kHz. Fig 1b shows an example of a filtered extracellular potential recorded at an active electrode site. Thresholds for spike detection were set individually for each channel to 5 times the standard deviation of the base electrode noise level. The standard deviation for each electrode was calculated over a 500 ms time interval.

Offline spike sorting was conducted prior to analysis to separate stimulation artifacts from neuronal signals at each electrode. Sorting was done using principle component analysis and a k-means algorithm. Identified signals were clustered together creating a neural assembly, or population response, at each electrode.

The cultured networks are spontaneously active and display a rich variety of firing behavior. A typical network has anywhere between 20–25 active electrodes, half of which display multiple units, with a spontaneous firing rate between 5–10 Hz. Fig 1c shows a raster plot of twenty seconds of spontaneous activity from nine active electrodes in a typical cortical MEA network. The black lines denote identified neuronal spikes recorded at each electrode.

### Stimulation

Electrical stimulation was applied to the culture through the electrodes using the commercially available stimulus generator STG 4002 (Multichannel Systems, Reutlingen, Germany). Stimulating electrodes were not used for recording and therefore excluded from analysis. Thirteen electrode stimulation sites were selected in an “L” shape configuration, consisting of two perpendicular rows of electrodes meeting at a point. Parameters for both the probing stimulation and the training signal described below were defined as in [13].

**Fig 2:**
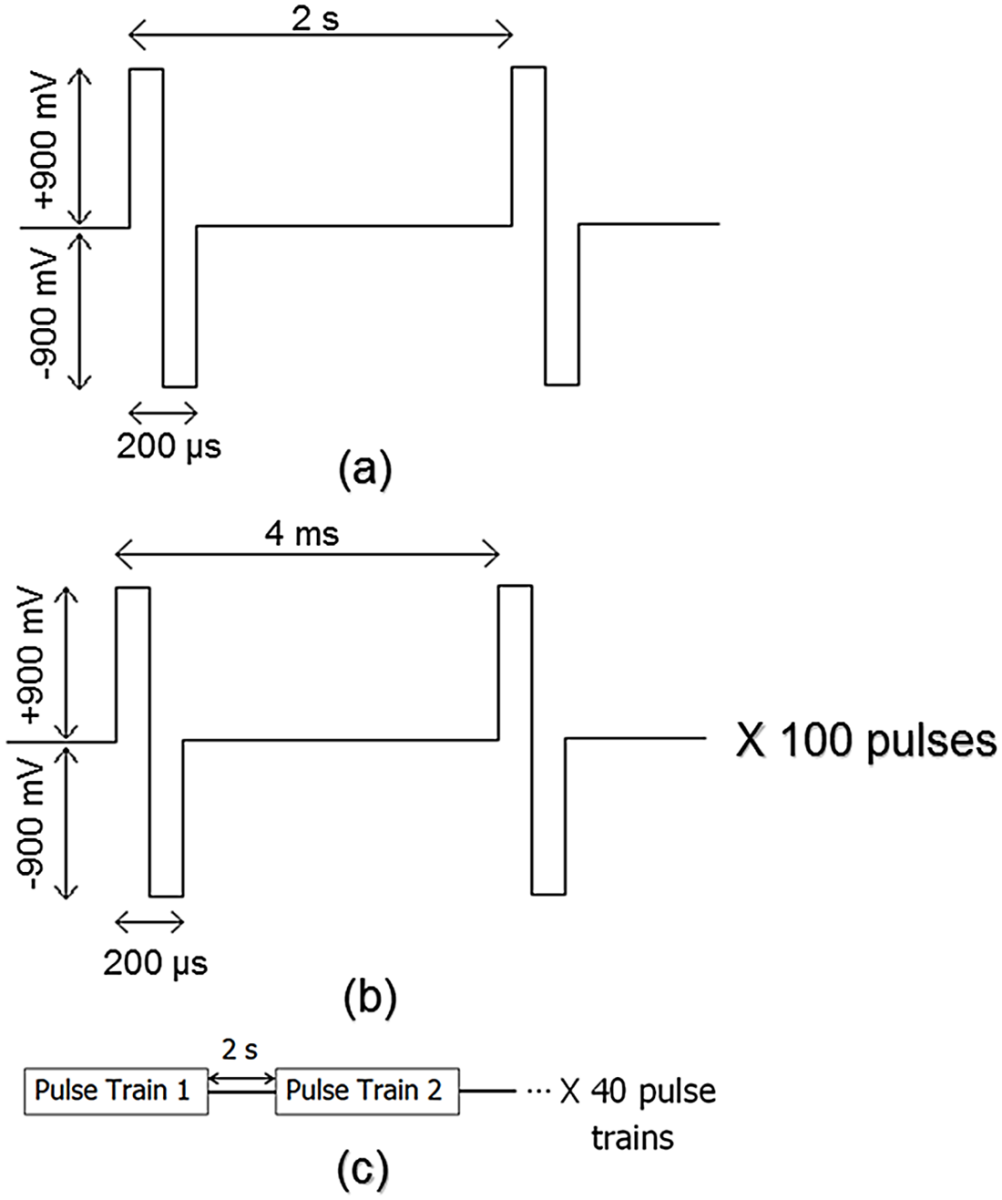
Parameters for probing stimulation and training signal. (a) Probing stimulation consisted of ± 900 mV bi-phasic pulses administered at a frequency of 0.5 Hz. Each phase lasted 100 *μ*s for total pulse length of 200 *μ*s. (b) Pulse trains consisted of 100, ± 900 mV bi-phasic pulses with frequency 250 Hz. (c) The training signal consisted of 40 total pulse trains administered once every two seconds.

Five minutes of spontaneous activity was recorded from each network prior to stimulation (baseline). Networks were then administered five minutes of a probing stimulation (pre-training) through the selected stimulation sites. Probing stimulation consisted of a 0.5 Hz biphasic pulse, 200 *μ*s pulse duration with 900 mV pulse amplitude. Fig 2a shows the parameters of the low frequency probing stimulation pulse.

After the pre-training stimulation phase, networks underwent a training procedure. The training signal (see Fig 2b and 2c), administered through the same electrodes as the probing stimulation, consisted of 40 pulse trains. Each train was comprised of 100 biphasic pulses with 4 ms between pulses, 200 *μ*s pulse duration and 900 mV pulse amplitude. These high frequency trains were delivered once every two seconds. Networks from *n* = 12 experiments received the training signal.

Once training was complete, five minutes of the probing stimulation (post-training) was re-administered to the networks. After the post-training stimulation phase, five minutes of spontaneous activity was recorded from the network (post-stimulation).

A separate group of networks were treated as controls to control for possible changes in network response as a result of natural fluctuations or system nonstationarity. These networks underwent the same experimental protocol described above, with the exception that they received a sham training period and did not receive any training signal. Networks from *n* = 10 experiments received the sham training and were kept as controls.

### Data Analysis

We examined the peristimulus time histogram (PSTH) for control and trained networks before and after the training period. Network activity was examined up to 50 ms after stimulus in 10 ms bins. The spike frequency in each bin was calculated individually for all active electrodes and averaged to give a network frequency per bin. In addition to spike frequency, spike reliability was calculated and averaged across all active electrodes to give a network spike reliability per bin. Spike reliability can be interpreted as the probability of seeing a network response to the stimulation, where a maximum value of 1 indicates that there was a response to every stimulus pulse. All analysis was done using code developed in MATLAB^*TM*^ (MathWorks, Natick, MA).

To account for the variability amongst networks within a group, each network’s post-training reliability and frequency were normalized with respect to their pre-training values. Any normalized value greater than 1 indicated an increase from pre to post-training and values less than 1 indicated a decrease. For example, we expected control networks to have normalized spike frequency and reliability values around 1 since they did not receive any training.

Prior to statistical analysis, normality of data was verified using Q-Q plots. Analysis of spike frequency and reliability between control and trained networks was carried out using a repeated measures one-way ANOVA with training (trained or control) as a between subject factor and time after stimulus (0–50 ms) as a within subject factor using SPSS (IBM, Armonk, NY). The Mauchly’s test was carried out to determine if sphericity was violated. A Greenhouse-Geisser correction was used if sphericity was violated and significance was determined as *p* < 0.05. If a statistically significant interaction was found, a Tukey’s post hoc analysis was run. All reported values are mean ± SEM.

### Pharmacology

To aid in our characterization of network response to stimulation, additional expeiments were implemented in which the network dynamics were pharamcologically altered with 6-Cyano-7-nitroquinoxaline-2, 3-dione disodium salt hydrate (CNQX, CAS 115066-14-3) and DL-2-amino-5-phosphonovaleric acid (APV, CAS 76326-31-3). CNQX and APV, AMPA and NMDA receptor antagonists respectively, were acquired from Sigma-Aldrich (St. Louis, MO). Their combined application to cell culture media has been shown to completely abolish spontaneous activity in cortical cell cultures [17]. Given their high solubility in water, stock solutions were prepared by dissolving them in cell culture media. All experiments were performed within one week, using the same stock solution, which was kept refrigerated until use. Solution was warmed to 37°C before each experiment. During the experiment, a bolus injection of CNQX+APV was applied to the 1 mL of cell culture media already in the culture, to a final concentration of 0.8 mM of AP5 and 80 *μ*M of CNQX [18]. Several networks (*n* = 4) were administered five minutes of probing stimulation before (pre-drug) and after (post-drug) application of CNQX+APV. Post-drug responses were normalized to pre-drug baseline response values and averaged across all networks.

### Connectivity Analysis

Analysis of network connectivity has become an important metric through which to study the dynamics of neuronal networks. In [19], the connectivity problem was addressed in a data assimilation framework utilizing recorded time series. More common though is analysis through spike train data. A variety of nonparameteric spike train methods have developed, ranging from standard cross-correlation to more advanced information theory approaches (see [20, 21] for interesting reviews of available methods). Of these techniques, transfer entropy has been well-documented and implemented as an effective tool for determining network connectivity [21, 22]. However, these spike train approaches are all limited by the need to define an arbitrary threshold with which to identify connections or non-connections within a network.

The Cox statistical test [23] as developed in [14] provided the first statistical technique for determining neuronal network connectivity from spike train data. The Cox method is a semiparametric approach for determining connectivity that assumes the interactions between neurons are represented by a proportional hazard model [14]. This method was further developed in [15] to provide a test for determining statistically significant changes in connection strengths over time.

A general description of the Cox method as implemented in this paper follows. For a more detailed description see [15]. The Cox method provides estimates of the network connection parameters $\hat{\beta }$, where ${\hat{\beta }}_{ij}$ denotes the estimated connectivity from neuron *i* to neuron *j*. In addition to estimate $\hat{\beta }$, the method calculates the covariance of $\hat{\beta }$. This allows for a statistical test for connectivity. We assume the null hypothesis *H*_0_: *β*_*ij*_ = 0, or that neuron *i* has no influence on neuron *j*. We reject this hypothesis if the confidence interval for ${\hat{\beta }}_{ij}$ does not include 0, meaning that it is a statistically significant connection. Assuming the Cox method is applied to multiple data recordings from the same network, we can detect changes in the connection strength from one recording period to the next. If the estimated ${\hat{\beta }}_{ij}$ lies outside the confidence interval of the previous period, then we say that the strength of the connection significantly changed.

## Results

### Networks that receive training display an increased response to probing stimulation

**Fig 3:**
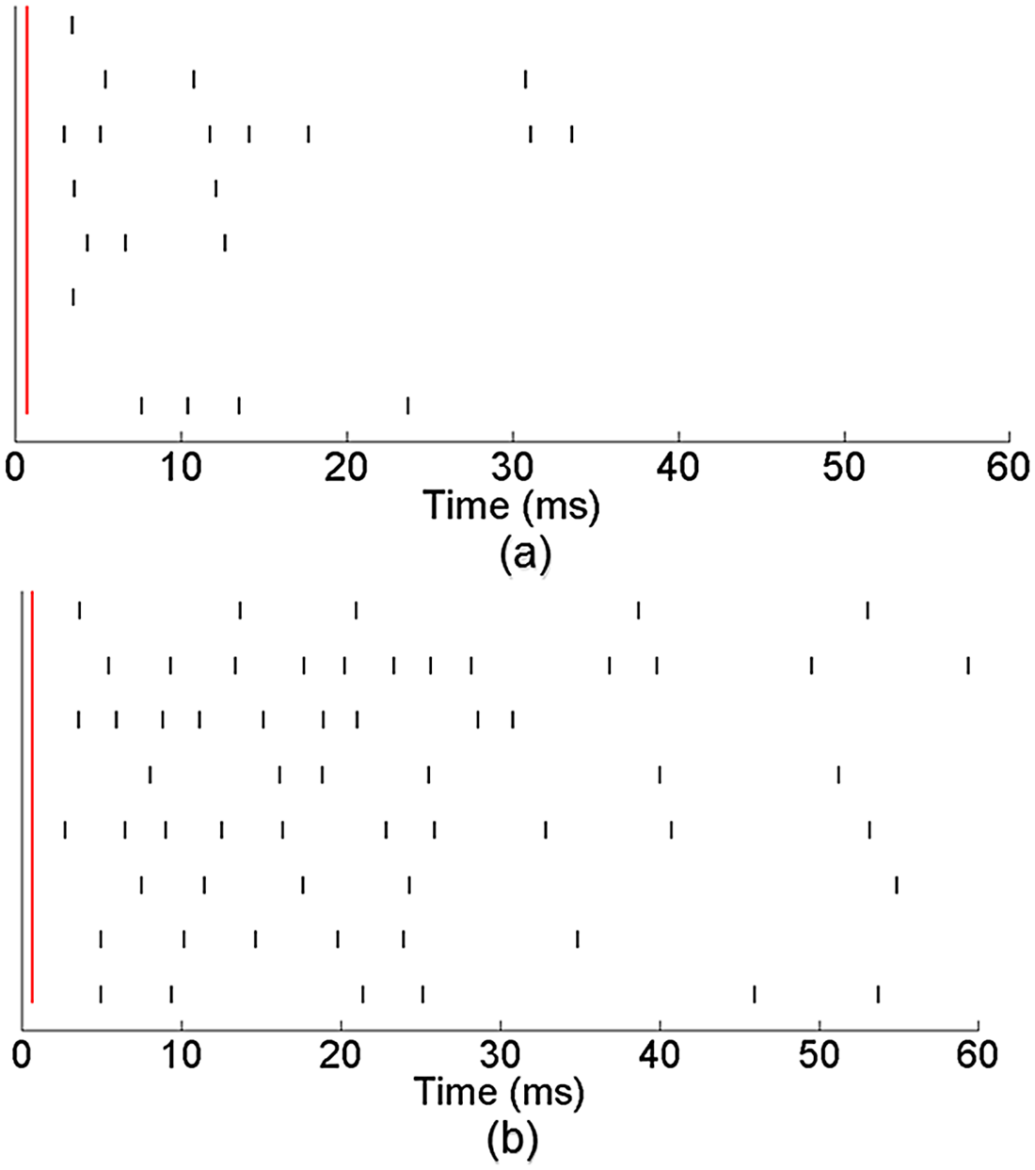
Network displayed an altered activity response to stimulation after the training period. Spiking activity from eight active electrodes from a representative MEA network is shown. Vertical red line indicates the time of the stimulus pulse and black lines denote neuronal spiking activity. Pre-training (a), we observe an immediate response across channels to the stimulus pulse. Post-training (b) the network continued to display the immediate response to the stimulation. However, it also exhibited a more prolonged activity response.

Fig 3 shows the activity response of eight active electrodes from a representative network that received training. The vertical red line indicates the time of a single stimulus pulse and the black lines denote neuronal activity in response to the stimulus. Fig 3a shows the network response pre-training and Fig 3b shows the response post-training. Pre-training the network exhibited an immediate response across channels to the stimulation within the first 20 ms after stimulus. However, the amount of activity after this initial response was inconsistent. Post-training, we observed a noticeable change in the network response. While the network still displayed the immediate response to the stimulus pulse within the first 10–20 ms, it also displayed a significant amount of activity 30–50 ms after stimulus.

**Fig 4:**
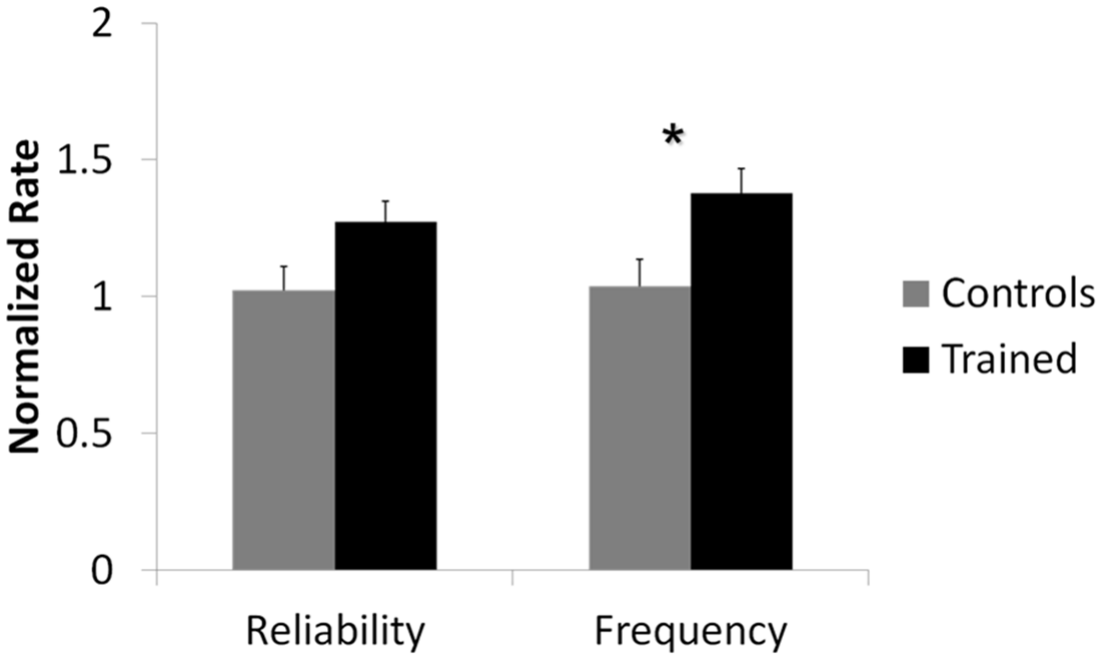
Network response to stimulation was significantly different for trained networks compared to controls. Mean normalized spike frequency and spike reliability over the first 50 ms after stimulation for networks that received training (*n* = 12) and networks that were kept as controls (*n* = 10) are shown. (*) denotes statistical significance *p* < 0.05. Analysis indicated a statistically significant overall difference in spike frequency between trained groups when compared to control groups. There was no statistically significant overall difference in spike reliability.

Results reported below are from *n* = 10 control and *n* = 12 trained networks. Fig 4 shows the overall effect over the first 50 ms after stimulation between networks that received training compared to networks kept as controls for both normalized spike frequency and normalized reliability. (*) denotes statistical significance of *p* < 0.05. Analysis revealed that there was a statistically significant overall difference in spike frequency between trained networks and control networks (*F*(1, 1.838) = 6.923, *p* < 0.05). Specifically, the mean normalized spike rate for trained networks was 1.377±0.090 and for control networks it was 1.024±0.099. This meant that post-training, networks that received the training signal responded to the probing stimulus with 37% more activity than they did pre-training. As expected, the response of control networks post-training did not differ greatly from their response pre-training. There was no statistically significant overall difference in spike reliability between the two groups.

### Increase in network response to probing stimulation is time-dependent

**Fig 5:**
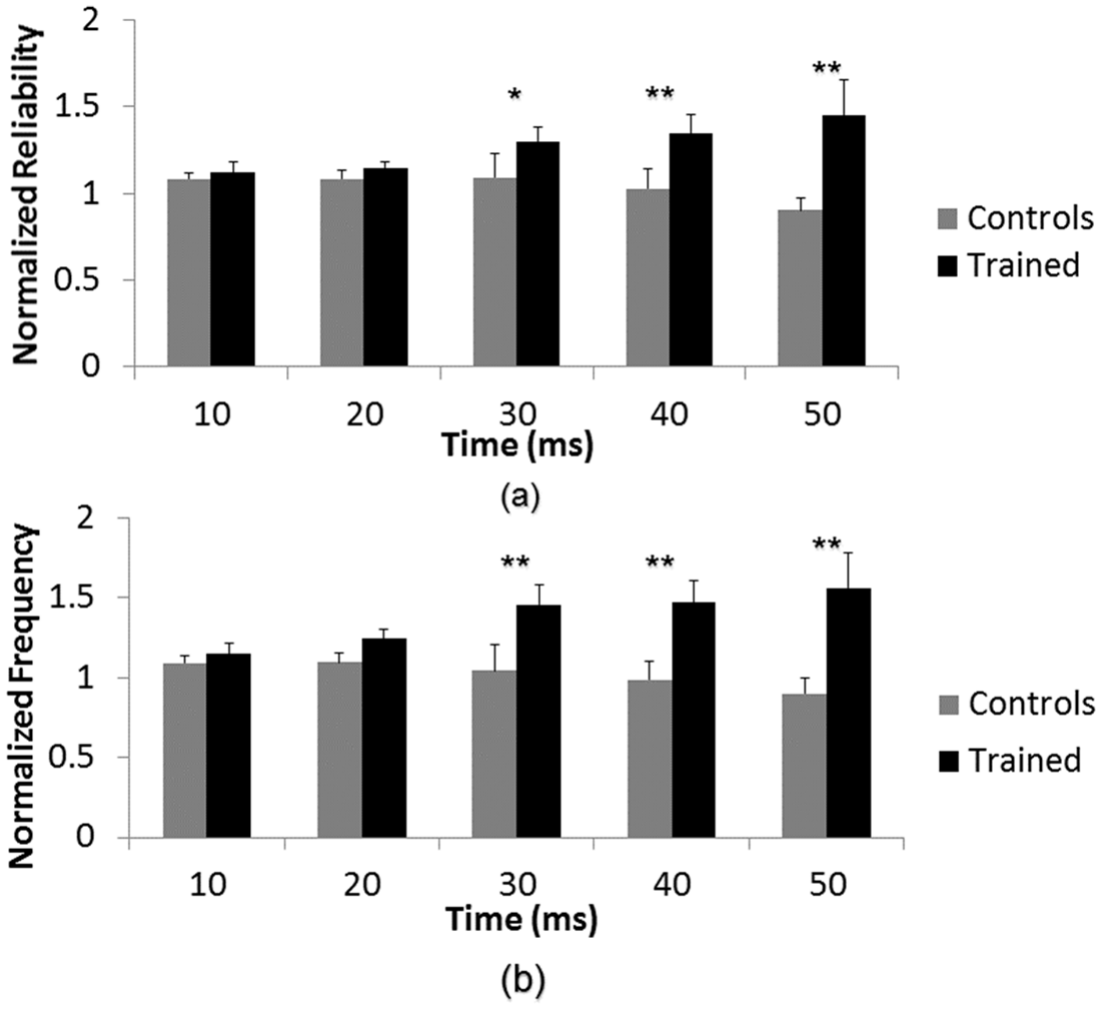
Effect of training is time-dependent. A statistically significant interaction between time after stimulus and training was found for both (a) spike reliability and (b) spike frequency (*p* < 0.05). A Tukey post-hoc analysis was run to determine the points of statistical significance. (**) denotes *p* < 0.01 significance and (*) denotes *p* < 0.05 significance. Post-hoc analysis showed that there was a statistically significant difference in normalized spike frequency and normalized spike reliability at time bins 20–30 ms, 30–40 ms and 40–50 ms after stimulus between trained and control networks.

Further analysis indicated a statistically significant interaction between spike frequency and time after stimulus(*F*(1, 1.838) = 4.290, *p* < 0.05) and between spike reliability and time after stimulus (*F*(1, 1.768) = 3.611, *p* < 0.05). Fig 5 shows the normalized frequency and reliability of control and trained networks as a function of time after stimulus. A Tukey post-hoc analysis was run to determine the points of statistical significance. This post-hoc analysis indicated a statistically significant difference between spike frequency of control and trained networks at time bins 20–30 ms (*p* < 0.01), 30–40 ms (*p* < 0.01) and 40–50 ms (*p* < 0.01) after stimulus. Reliability between control and trained networks was also statistically different at time bins 20–30 ms (*p* < 0.05), 30–40 ms (*p* < 0.01) and 40–50 ms (*p* < 0.01).

There was approximately a 50% increase in spike frequency, as well as a 30–50% increase in spike reliability for trained networks in the range of 20–50 ms after stimulus. This large increase in network response, in addition to the delayed effect, suggests that the training fundamentally changed the network dynamics.

**Fig 6:**
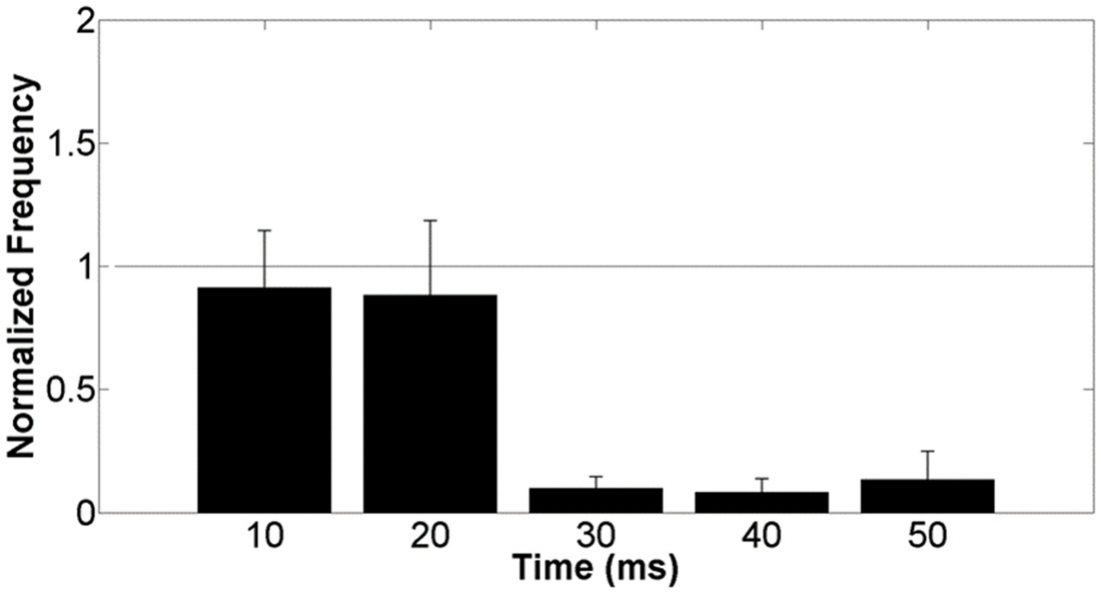
Response to stimulation consists of two phases. Networks receiving CNQX+APV treatment exhibited a substantial decrease in response 20–50 ms after stimulation as compared to pre-drug baseline values. The initial response period of 0–20 ms remained relatively unchanged. This would imply that the network response is comprised of two phases, the later of which is governed synaptically. This suggests that the observed enhancement is the result of synaptic potentiation.

To investigate the nature of these changes, an additional group of networks (*n* = 4) were treated with a combination of CNQX+APV. Fig 6 shows the mean normalized spike frequency of these networks over the first 50 ms after probing stimulus. Application of CNQX+APV results in a clear decrease in network response to stimulation as compared to pre-drug baseline values during the time frame of 20–50 ms after stimulus. There was no noticeable difference in network response 0–20 ms after stimulus. These results indicate that the later portion of the stimulus response is driven synaptically, whereas the initial phase is not. This would therefore suggest that the increased response observed in Fig 5 is the result of synaptic enhancement.

### Identifying network connectivity

To our knowledge there has been no direct comparison between the Cox method and transfer entropy for determining network connectivity. Here we briefly evaluate the performance of both methods in networks of Izhikevich neurons. We investigated several versions of transfer entropy, in particular exploring higher order and delayed versions as described in [21]. For the simulations here, we found it optimal to set order to 3 and delay to 1. A more detailed comparison in terms of method-specific advantages and disadvantages is left for a later time.

The Izhikevich system [24] is a two-dimensional model known for its combination of biophysically plausible neuron firing dynamics with computational simplicity. It is described by the equations

$$\begin{array}{ccc} \frac{dv}{dt} & = & 0.04v^{2}+5v+140-u+I\\ \frac{du}{dt} & = & a(bv-u) \end{array}$$ (1)

where *v* is the membrane potential and *u* represents a recovery variable. Once a neuron’s spike reaches a peak of +30 mV, *v* and *u* are reset according to the following:

$$\begin{array}{c} \text{if }v\geq \text{30 mv then }\left\{ \begin{array}{l} c\to v\\ u+d\to u \end{array}\right. \end{array}$$ (2)

The parameters *a*, *b*, *c*, *d* are set in the model to generate different neuron firing behaviors. *I* corresponds to the synaptic current received as a function of the network connectivity.

In our comparison, we considered random networks with a “mixed population” of Izhikevich neurons consisting of regular spiking, fast spiking, intrinsically bursting and chattering neuron firing behaviors. These heterogeneous networks were generated at increasing levels of connectivity. For example, a network of 10 neurons has 90 possible connections (ignoring the possibility of self-connections). At a connectivity level of 20%, that implies there are 18 actual connections within the network. As the connectivity level increases resulting in a larger number of network connections, the identification problem becomes increasingly difficult as the potential for transitive connectivity becomes higher.

In evaluating the performance of these methods, specificity (true negative rate) and sensitivity (true positive rate) statistics were calculated. Specificity can be thought of as number of correctly identified non-connections and sensitivity as the number of correctly identified connections. Since the Cox method is a statistical test, it can be implemented a priori with a pre-defined confidence level. For the results here, we chose a confidence level of 95%, meaning the resulting specificity of the results was at least 0.95. Transfer entropy on the other hand is a non-statistical method and requires an ad hoc threshold to be set. To facilitate a direct comparison, thresholds were chosen such that the resulting specificity of the transfer entropy results was at least 0.95. As such the sensitivity of both methods, or the number of correctly identified connections, could be compared.

**Fig 7:**
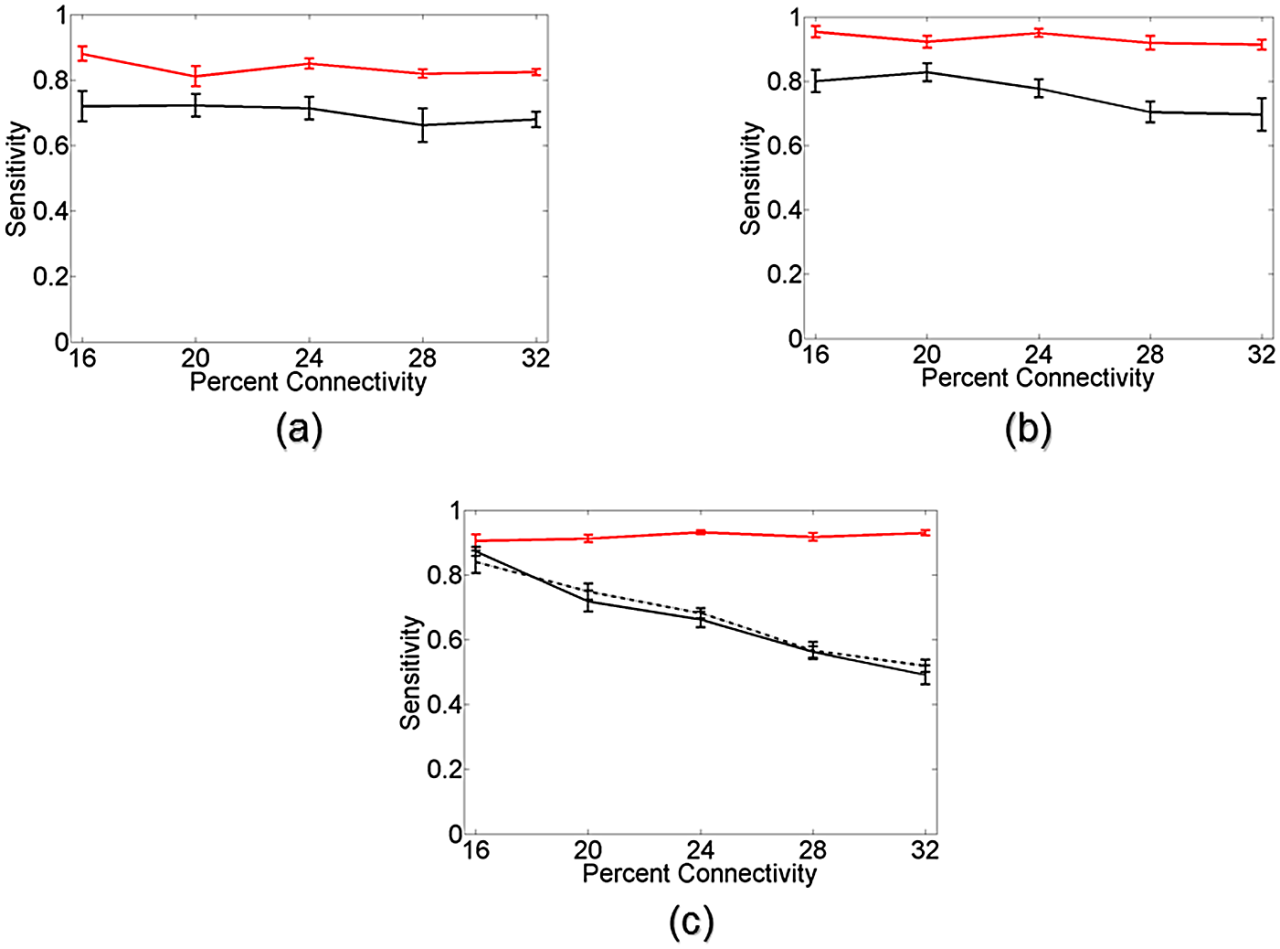
Connectivity comparison in networks of Izhikevich neurons. Sensitivity, or number of correctly identified connections, for the Cox method (red curve) and transfer entropy (black curve) in heterogeneous networks of Izhikevich neurons at varying levels of connectivity. Error bars denote standard error over 10 random network realizations. (a) Results for networks of 10 neurons when approximately 500 spikes per neuron are available for analysis. (b) Results for networks of 10 neurons when approximately 1000 spikes per neuron are available. While both methods receive an expected increase in performance going from 500 to 1000 spikes, there is a clear performance advantage in using the Cox method over transfer entropy in both the 500 and 1000 spikes per neuron situation. (c) The discrepancy in performance is even more evident when examining networks of 20 Izhikevich neurons. The Cox method (red curve) with access to only 1000 spikes per neuron outperforms transfer entropy with access to 2000 spikes per neuron (black curve) or 4000 spikes per neuron (dotted black curve).

Fig 7 shows the sensitivity results of the Cox method (red curves) and transfer entropy (black curves) as a function of network connectivity for various networks of Izhikevich neurons. Error bars denote standard error over 10 random network realizations. Fig 7a and 7b shows the results of both methods in networks of 10 neurons with approximately (a) 500 spikes per neuron and (b) 1000 spikes per neuron. For both methods there is an improvement in connectivity identification as the number of spikes per neuron increases, which is to be expected. However, the Cox method displays a clear performance advantage over transfer entropy in both situations. This advantage becomes even more evident when examining the connectivity problem in networks of 20 neurons. Fig 7c shows the results of the connectivity identification in networks of 20 neurons. The Cox method (red curve) using only 1000 spikes per neuron is able to identify the majority of the network connections. Transfer entropy, using 2000 spikes per neuron (black curve) and 4000 spikes per neuron (black dotted curve), has a difficult time identifying the network connectivity given the limited number of spikes per neuron in these larger networks.

While there is a clear performance advantage of the Cox method over transfer entropy in the examples considered, it should be noted that transfer entropy allows for greater scalability in terms of network size. The computational demands of the Cox method can make its implementation in larger networks difficult. As previously mentioned, a more thorough analysis of the advantages and disadvantages of both methods as well as their performance under various conditions should be explored.

### Training results in significant changes in network connection strengths

We explored the possibility of network connection strength changes caused by the training signal using the Cox connectivity method. We applied the Cox method to the baseline and post-stimulation periods of the experiment. During these experimental periods there was no external stimulation applied to the network meaning that all neuronal activity was the result of the network’s natural spontaneous firing dynamics. To ensure that there were stable connection estimates within each period, the baseline and post-stimulation periods were each divided into two sub-periods. Specifically we labeled these as B1:B2 during baseline and P1:P2 during post-stimulation. The Cox method was applied to each of these periods, resulting in a total of four estimates for each connection parameter.

**Fig 8:**
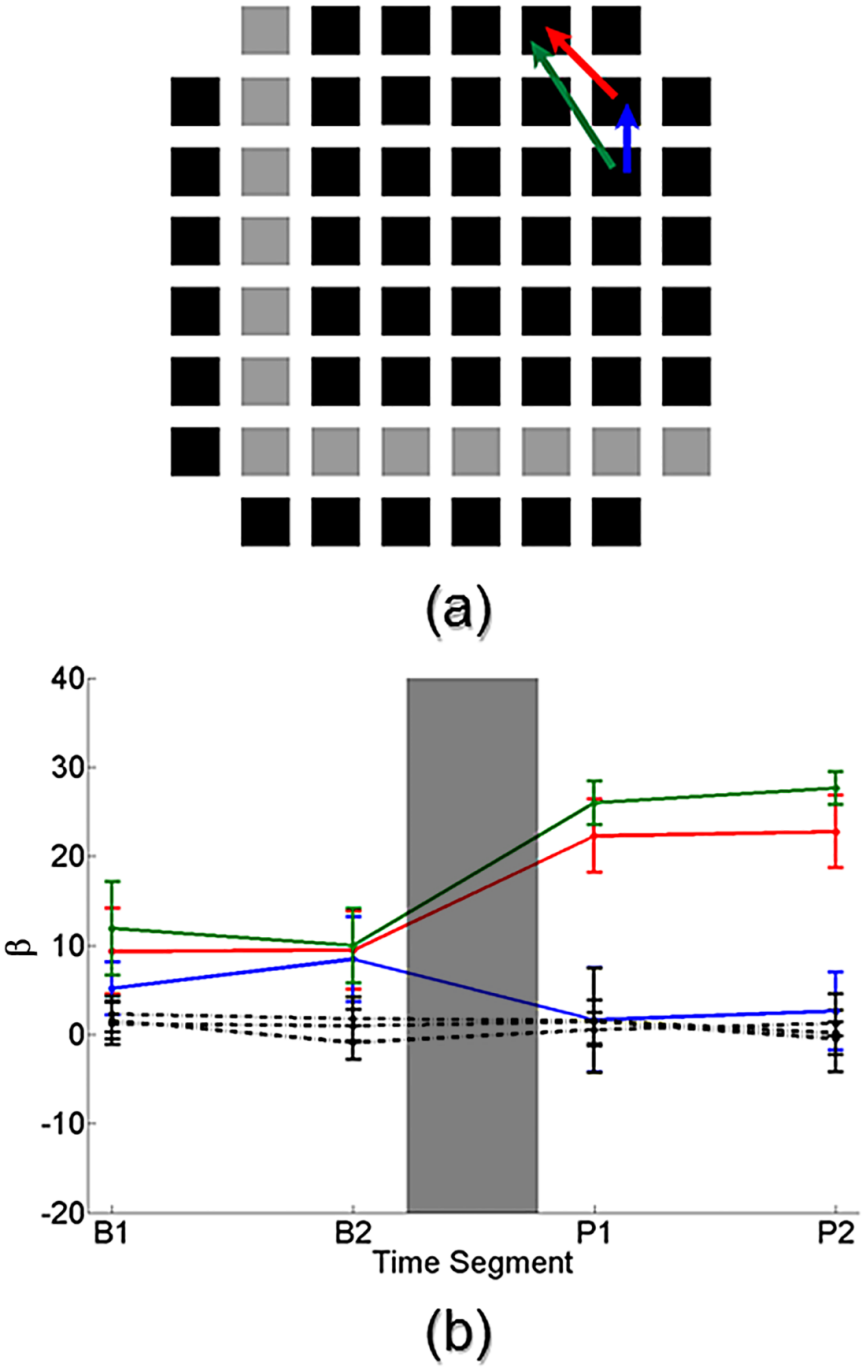
Network connectivity analysis of a representative trained network. (a) Pictorial representation of a network that received training. Squares indicate individual electrodes from an MEA where black squares represent electrodes available for recording and gray squares indicate electrodes used for stimulation. Three statistically significant connections identified by the Cox connectivity method during baseline recordings are drawn (red, green and blue arrows). Arrowhead denotes direction of the connection. (b) The estimated strength of several connection parameters from the MEA network in (a) are shown. Error bars denote 99% confidence interval and gray box signifies the time during which training was administered. Solid colored connections correspond to those drawn in (a). The three connections undergo a significant change in connection strength after the training, namely the red and green connections increase in strength and the blue connection decreases in strength. Several non-connections (dotted black lines) are also shown whose strength does not change over time.

Fig 8a shows a pictorial representation of an MEA network that received training. Each square represents one of the array’s electrodes, where black squares denote electrodes available for recording and gray squares indicate electrodes used for stimulation. Three network connections identified by the Cox method as statistically significant during the baseline period are shown, represented by the red, green and blue arrows. The arrowhead indicates the direction of the connection. Fig 8b shows the estimated strength of several connection parameters from the network in Fig 8a as a function of baseline and post-stimulation periods. Error bars denote the estimated 99% confidence interval and the gray box represents the administration of the training signal to the network.

The solid colored connection parameters correspond to those shown in Fig 8a. For all three of these connections the estimated strengths were consistent within a period, namely there were no statistically significant changes in connection strength from sub-periods B1 to B2 and from P1 to P2. This was to be expected given that there was no stimulation or other experimental manipulation between B1 and B2. However between the periods, namely from B2 to P1, all three connections undergo statistically significant changes in their estimated connection strengths. The red and green connections experience a significant increase in their strength, while the blue connection has a significant decrease in its strength. Additionally, several non-connections (black dotted lines) are shown over the four periods. The estimated strengths of these non-connections remain consistent throughout the four periods, further reinforcing the strength changes that we were able to find.

Our connectivity analysis found a range of identified connections across networks. Of note, the significance of several connections changed across periods as a result of identified strength changes. Considering only those connections that were consistently found as significant during both periods, on average the Cox method determined that approximately 37% of possible network connections were significant. Of these consistent connections that underwent a signficant change in strength, approximately 60% increased in strength and 40% decreased in strength. However as previously mentioned, there were additional strength changes within the network that resulted in connections with varying significance.

## Discussion

The response of the neuronal networks to electrical stimulation can be broken down into multiple phases, allowing for a more thorough analysis of the changes observed in the network response dynamics. The initial phase of network activity within the first 20 ms after stimulus corresponds to an early response dynamic that is directly caused by the stimulation [1, 18, 25–28]. This direct response to the stimulation is driven electrically and not synaptically [25, 28] and is likely the result of electrical excitation of nearby axons [1, 18]. In the second phase, a burst of activity across the network occurs between 20–200 ms after stimulation [25–28]. This phase of the activity response relies on synaptic transmission [28].

Our results indicated that networks receiving the high frequency training signal developed a stronger response to the low frequency probing stimulation. This increased response was overall statistically significant as compared to control networks which received no training. Additionally, a statistically significant interaction effect was found between time after stimulus and whether or not a network received training. Follow-up post-hoc analysis indicated that networks receiving the training signal exhibited a statistically significant increase in spike frequency and spike reliability in the range of 20–50 ms after stimulation as compared to controls. This time window corresponds with the network response dynamics that are driven synaptically. This observed increase would therefore suggest that the effect of the training signal could be synaptic potentiation. The lack of a statistically significant difference between control and trained networks up to 20 ms after stimulus further supports the idea of synaptic potentiation, as the network response during this time is driven directly by the electrical stimulation.

The subsequent connectivity analysis of spontaneous activity from a trained network revealed connections whose estimated strength significantly increased as a result of the training. However, there were also connections whose estimated strength did not change or decreased as the result of the training. As detailed by Jimbo et. al. [29], high frequency stimulation (or tetanization) can lead to a simultaneous potentiation and depression in networks. In [13], the authors noted that by increasing the number of stimulating electrodes they increased the likelihood of inducing potentation. However, even with using more than 10 stimulating electrodes the authors noted instances where a depressed response within the networks occurred.

Our findings in cortical networks were somewhat consistent with those found in [13] for hippocampal cultures. However, the additional time-dependent effects shown here further supports the idea that the training resulted in a form of synaptic potentiation. Subsequent connectivity analysis revealed identified network connections whose strength both increased and decreased. This invites the possibility of local effects within the network that should be further explored.

The results presented here open up several areas that require further investigation. Naturally, the duration of the observed enhancement needs to be explored. This would allow us to determine whether or not the training-induced potentiation was short-term or long-term. We view the Cox method as integral to this examination as it would allow us to track the changes in connection strengths across days. Furthermore, additional study is required to identify the specific cellular mechanisms involved in the training enhancement. By blocking specific cellular receptors (e.g. NMDA receptors) and repeating the stimulation paradigm described here, we would be able to identify the mechanisms involved in the process. We are currently pursuing this investigation in cultures treated with NMDA antagonists.
